# [Expression of Concern] Rapamycin-induced miR-30a downregulation inhibits senescence of VSMCs by targeting Beclin1

**DOI:** 10.3892/ijmm.2026.5981

**Published:** 2026-09-07

**Authors:** Pan Tan, Haiqin Wang, Junkun Zhan, Xinyu Ma, Xingjun Cui, Yanjiao Wang, Yi Wang, Jiayu Zhong, Youshuo Liu

Int J Mol Med 43: 1311-1320, 2019; DOI: 10.3892/ijmm.2019.4074

Following the publication of the above paper, it was drawn to the Editor's attention by an interested reader that a pair of unsuitable antibodies may have been used as a part of the western blot analysis shown in Fig. 1E on p. 1314. First, the antibody with the catalogue number ab51243 (purchased from Abcam), is not specific for p16-INK4a (the senescence- associated protein studied in this paper), but a totally different protein with a similar name, p16-ARC. A concern was also raised regarding the anti-β-galactosidase antibody (cat. no. ab9361; Abcam) that was employed, which is specific for the bacterial protein and is only distantly related to human β-galactosidase according to its sequence (neither is this antibody the closest homologue to bacterial β-galactosidase in the human proteome).

The authors have been contacted by the Editorial Office to offer an explanation for the selection of these apparently unsuitable antibodies for the western blot analysis, and we are awaiting their response. Owing to the fact that the Editorial Office has been made aware of a potential problem surrounding the design of these experiments in this study, we are issuing an Expression of Concern to notify readers of this issue while the Editorial Office continues to investigate this matter further.

